# Run or Die: A Didactique Case Report of a Rare Cause of Lactic Acidosis in Emergency Medicine

**DOI:** 10.1155/2020/5671296

**Published:** 2020-10-24

**Authors:** Jean-Baptiste Bouillon-Minois, Jeannot Schmidt, Frédéric Dutheil

**Affiliations:** ^1^Clermont Auvergne University, CNRS, LaPSCo, Physiological and Psychosocial Stress, University Hospital of Clermont-Ferrand, CHU Clermont-Ferrand, Emergency, F-63000 Clermont-Ferrand, France; ^2^Clermont Auvergne University, CNRS, LaPSCo, Physiological and Psychosocial Stress, University Hospital of Clermont-Ferrand, CHU Clermont-Ferrand, Preventive and Occupational Medicine, WittyFit F-63000 Clermont-Ferrand, France

## Abstract

**Introduction:**

Acidosis with traumatic brain injury is a common and serious cause of consciousness disorders in emergency medicine. Extreme acidosis is significantly associated with high mortality (more than 67% if pH levels are under 7). *Case Presentation*. We describe the case of a 23-year-old man with unknown medical history who was found near the entrance of the emergency department sweat with a tachypnea (55 per minute), a lot of blood around him, and confused. The initial hypothesis was a hemorrhagic shock after a fight, but he did not have any hemodynamic trouble. The initial venous gazometry showed a major lactic acidosis (pH less than 6,8, HCO3 incalculable and lactate up to 20 mmol/L). A Focused Assessment with Sonography in Trauma-echography (FAST-echo) and secondly a body-tomodensitometry were conducted and did not reveal any anomaly. The team was now thinking that the patient situation was caused by an epileptic seizure (association of lactic acidosis and confusion), and the bleed was a consequence of the head trauma. The patient was treated only by NaCl 0,9%. One hour after his admission, the tachypnea began to decrease and he could speak and explain what was happen. He had to run as fast as possible to escape to a fight. The last gazometry, realized 2 hours after his admission, finds a normal pH at 7,35, HCO3 24,5 mmol/L and lactate 2,6 mmol/L. He was authorized to going home.

**Conclusion:**

We report here a rare case of major lactic acidosis in emergency medicine caused by a supramaximal effort.

## 1. Introduction

This report highlights for a common situation in the Emergency Department (a patient with unknown medical history who obviously had a traumatic brain injury and lactic acidosis without apparent cause) but a rare aetiology of acidosis in emergency medicine, which is of special interest because a very few emergency physicians or intensive care anaesthetist could have thought of this pathology.

## 2. Case Presentation

A 23-years-old black man was found near the entrance of the emergency department with lots of blood around him. He was confused and sweating heavily. The initial vital parameters found were a sinusal tachycardia of 140 per minute, a good blood pressure of 125/75 mmHg, a normal SpO2 of 96%, a significant tachypnea of 55 per minute, and a temperature of 37.2°C. The initial hypothesis was a hemorrhagic shock after a major trauma. The initial venous gazometry showed a major lactic acidosis (Table 1) that was monitored 5 minutes later by an arterial exam. In order to intubate, he was preoxygened with a 15 L/min mask and an intravenous injection of NaCl of 500 mL at 0.9%. A Focused Assessment with Sonography in Trauma-echography (FAST) was conducted and did not reveal any anomaly. During this FAST, his head wound was fixed with 15 staples. We decided to sedate him with 4 mg of Midazolam intravenous to conduct a body-TDM that did not reveal any anomaly that could have explained the patient's condition. The team was now thinking that the patient situation was caused by an epileptic seizure (association of lactic acidosis and confusion) consecutive to the head trauma. One hour after his admission, his tachypnea began to decrease. The patient then explained that he had had an altercation, had been dragged in a building hall, and beat out for several minutes. He succeeded to escape and began to run for one kilometer as fast as he could, and eventually arrived at the entrance of the emergency department.

We analyzed his gazometry twice: one and two hours after his admission. Table 1 shows an acute decrease of the lactic acidosis. The patient was asymptomatic 2 hours after and went back home.

## 3. Discussion

On one hand, traumatic brain injury is a common cause of consciousness disorders in emergency medicine. On the other hand, extreme acidosis is significantly associated with high mortality. In a French hospital, it has been shown that 67.5% of patients who died in the Intensive Care unit had pH levels lower than 7 in the 24 hours that followed their admission [1]. Similar levels were reported in an Israeli hospital [2]. Furthermore, a significant link was found between venous lactate elevation and mortality with suspected infection in patients in emergency department [3, 4].

Lactate levels are extremely studied in sports medicine. A relation between lactate level and possibilities to have a professional ski career has been studied in Austria [5]. Lactate level is used to study 5 maximal graded exercise tests to evaluate the best one [6] and in 400 m track runners and found that it was, on average, 20 mmol/L [7]. Fortunately, lactate level in sports medicine are not associated with higher mortality.

To conclude this case, before the patient could speak, we did not envisage the supramaximal exercise in the emergency department because of the scarcity of this diagnosis and the presence of a common differential diagnosis. The fast decrease of lactate level that occurred was of great interest here. However, we do not think that supramaximal effort must be evoked as first line with a similar case.

## Figures and Tables

**Table 1:** Cinetic of gazometry parameters.

	Time (minutes)			
	t0	t5	t60	t120
Type of gazometry	Venous	Arterial	Venous	Venous
pH	<6.80	<6.80	7.21	7.35
PCO_2_ (mmHg)	31	21	40	42
PO_2_ (mmHg)	83	267	22	28
Na (mmol/L)	149	141	136	135
K (mmol/L)	3.5	3.5	4.3	3.9
Lactate (mmol/L)	>20	>20	10.5	2.6
HCO_3_ (mmol/L)	Incalculable	Incalculable	17.2	24.5
Hb (g/dL)	17.4	17	14.8	15
